# Effects of Neonicotinoid Pesticide Metabolic Compounds on Medaka (*Oryzias latipes*) Embryo Development

**DOI:** 10.3390/biology12121460

**Published:** 2023-11-23

**Authors:** Hotaka Kai, Arisa Mita, Masahiro Yamaguchi

**Affiliations:** Department of Chemistry and Biochemistry, National Institute of Technology (KOSEN), Suzuka College, Shiroko-Cho, Suzuka 510-0294, Mie, Japan

**Keywords:** neonicotinoid metabolites, medaka embryo, developmental anomaly, thrombus

## Abstract

**Simple Summary:**

Pesticides are essential for safeguarding food resources. By using pesticides to kill insects that harm crops, crop yield (quality and quantity) is improved. However, pesticides that remain in the soil and aquatic environment must not have a negative impact on living organisms. In this study, medaka eggs were exposed to high concentrations of pesticide-like compounds and the resultant effects were examined. Medaka eggs exposed to high concentrations of pesticide-like compounds had collapsed eyes and abnormal blood flow. In addition, sudden death just before hatching was observed. This study elucidated the effects of high concentrations of pesticide-like compounds on the development of medaka eggs. Further, we obtained new knowledge about the risks of pesticide use.

**Abstract:**

Neonicotinoids, including imidacloprid, are pesticides that resemble nicotine and undergo slight chemical alterations through metabolic changes in the environment. However, the effects of these metabolites on organisms remain unknown. In this study, we assessed the developmental processes of medaka embryos exposed to neonicotinoid metabolites. The target compounds were imidacloprid metabolites: 2-chloro-5-pyridine carbaldehyde (CPC) and 6-chloronicotinic acid (6-CNA). Medaka embryos within 6 h of fertilization were exposed to the compounds, and their developmental processes were observed under a stereomicroscope. Medaka embryos exposed to 5 mg/L CPC showed no abnormalities compared to the controls. Contrastingly, medaka embryos exposed to 10, 15, and 20 mg/L CPC showed abnormalities such as thrombus formation, asymmetry, disorganized development of the eyeballs, and low blood flow. This trend was more pronounced at higher CPC concentrations. On the other hand, embryos exposed to 80 and 160 mg/L 6-CNA showed no abnormalities until day 7 of exposure. However, on day 8 of exposure, sudden embryo death was observed. Both compounds may have bound to acetylcholine receptors as agonists; however, their effects were different. CPC caused abnormal development and 6-CNA caused inhibition of hatching gland development and/or synthesis of the hatching enzyme.

## 1. Introduction

Insecticides containing neonicotinoid are widely used to exterminate harmful insects from agricultural products as well as lice and fleas on pets [1,2,3]. Seven types of neonicotinoid insecticides are commonly used for these purposes: acetamiprid, imidacloprid, clothianidin, dinotefuran, thiacloprid, thiamethoxam, and nitenpyram. These substances are water-soluble and are absorbed through the roots of plants, spreading throughout the plant body to the leaf tips and protecting the entire plant from insects [4]. Neonicotinoids can bind to and activate nicotinic acetylcholine receptors as agonists, thereby affecting neurotransmission and the formation of a neural circuit [5]. Because of their higher affinity for insect nicotinic acetylcholine receptors than their mammalian counterpart, they have been thought to be relatively harmless to mammals or vertebrates compared to organophosphorus pesticides [6].

Neonicotinoid insecticides have been on the market since the 1990s, and their demand has continued to grow, exceeding EUR 600 million in 2006 [7]. However, neonicotinoid insecticides were reported to damage non-target organisms. Henry et al. [8] and Whitehorn et al. [9] reported that neonicotinoids negatively affect honeybee migration, colony development, and queen survival. This raised global concerns about the effects of neonicotinoids on honeybees. In addition, the European Food Safety Authority published a report evaluating the effects of neonicotinoid pesticides on honeybees [10]. Following the report, the use of imidacloprid, thiamethoxam, and clothianidin was temporarily banned in the EU in 2014. Subsequently, the outdoor use of these three neonicotinoids was formally prohibited in 2018 [11]. To this end, various countries around the world, including the European continent, have developed policies to prohibit the use of neonicotinoids. For example, in 2018, the Turkish government issued an emergency order banning the use of three neonicotinoid pesticides, including imidacloprid, in connection with mass honeybee deaths [12]. Fiji banned imidacloprid use in January 2020 [13]. In the U.S. state of Washington, a pollution prevention agreement was signed which bans the use of imidacloprid in oyster farms [14]. However, in some countries, the emergency use of these neonicotinoids was requested and approved for insect control in outbreaks. It is therefore clear that the use and regulation of pesticides is a very difficult issue.

As mentioned above, neonicotinoids have been believed to be relatively safe in mammals due to their selective toxicity to insects. However, Kimura-Kuroda et al. [15] found that acetamiprid and imidacloprid induced excitatory responses in developing cultured rat neurons at concentrations as low as 1 µM via nicotinic acetylcholine receptors and expressed concern about the adverse effects of neonicotinoids on brain development in children. In addition, using cultured neonate rat cerebellum, they found that exposure to acetamiprid and imidacloprid caused a slight disturbance in Purkinje cell dendritic arborization and disorganized gene expressions [16]. Takada et al. [17] also reported that dinotefuran inhibited neurotransmission and stress hormone secretion in rats based on three behavioral tests and immunohistochemical observations. The results of these studies indicate that neonicotinoids have diverse effects on the nervous and immune systems in mammals. Owing to this, the interest in neonicotinoids has been increasing globally [18]. Based on studies that report the negative impacts of neonicotinoids on mammals, the European Food Safety Authority officially stated that acetamiprid and imidacloprid affect the developing human nervous system and proposed to set more conservative values of accepted daily intake and acute reference doses for these two neonicotinoids in 2013 [19].

However, shipments of neonicotinoid insecticides into Japan in 2018 were as follows: acetamiprid 50.2 t, imidacloprid 67.5 t, clothianidin 74.8 t, dinotefuran 167.0 t, thiacloprid 14.2 t, thiamethoxam 46.1 t, and nitenpyram 5.6 t [20]. In addition, unlike in Europe, a neonicotinoid prohibition policy has not been officialized in Japan. Contrarily, residue limits of acetamiprid, clothianidin, and thiamethoxam have recently been relaxed. The re-evaluation of toxicity of five neonicotinoids (acetamiprid, imidacloprid, clothianidin, dinotefuran, and thiamethoxam) is scheduled under the revised Pesticide Control Law [21]. However, it is unclear whether the re-evaluation will result in tighter regulation of neonicotinoids in Japan. As mentioned above, neonicotinoids are water-soluble, thus neonicotinoids applied in the field can easily migrate to the aquatic environment. In particular, neonicotinoids have been detected in Japanese rivers [22] and marine regions [23,24]. Furthermore, it should be noted that neonicotinoids gradually change their chemical structure in soil and aquatic environments. For example, the chemical structure of imidacloprid in the aqueous environment is gradually altered upon light irradiation to 2-chloro-5-pyridine carbaldehyde (CPC), desnitroimidacloprid (DNI), or 5-(aminomethyl)-2-chloropyridine (AMCP) and finally to 6-chloronicotinic acid (6-CNA) [25]. These transformed products are called pesticide transformation products in water environments (PTPWs), and they retain the structure of the halopyridine (toxicophore) which imparts toxicity as in imidacloprid. Therefore, to formulate a use policy and to establish residue limits of neonicotinoids, the toxicity of these PTPWs, as well as primary chemicals, should also be evaluated.

In this study, we evaluated the impact of PTPWs of imidacloprid, CPC, and 6-CNA on the development of medaka embryos. Medaka is a Japanese common freshwater fish and is used as a target fish for toxicity tests. Reducing insecticide toxicity is one of the high-priority targets in the quest for sustainable agriculture. The evaluation of PTPWs in this study will contribute to formulating precise policies on pesticide use, thus protecting aquatic organisms from toxic pesticides, which is important for environmental sustainability. The findings will assist in clarifying the effects of neonicotinoid PTPWs, providing necessary information for risk management of chemical substances. For people to lead convenient and comfortable lives and to achieve sustainable development, it is necessary to ensure the safety and security of the chemicals around us. The results of this research can reveal the risks of indispensable pesticides to the ecology, which is cardinal, for example, in the field of agriculture, in turn necessitating sustainable development. Identifying this risk is directly related to safety and security. Therefore, we conducted a study to clarify the biological risks of pesticides using medaka, one of the bioassay model organisms used worldwide.

## 2. Materials and Methods

### 2.1. Test Fish

The test fish, medaka (*Oryzias latipes*), were procured from the National Institute for Environmental Studies (Tsukuba, Japan) and used as the parent fish. Mature medaka were kept in a 45 L tank at a density of approximately 30 fish (male: 10, female: 20) in a water temperature of 25 °C with a 16 h light: 8 h dark cycle. The medaka were fed *Artemia salina* and an artificial feed Otohime Β2 (Nisshin Marubeni Feed) once daily. The water in the tanks was changed every 2–3 days to maintain sanitary conditions. The dechlorinated water used as medaka breeding water had a pH of 6.8–7.5, an electrical conductivity of 16.6–17.3 mS/m, and a total hardness of 50 mg CaCO_3_/L. Female parent fish were scooped with a net, and embryos attached to their bellies were collected by brushing, transferred to Petri dishes, and stored in a thermostatic chamber at 25 °C. Medaka embryos were defined as day 0 embryos on the day the parent laid eggs. Embryos up to 6 h after fertilization were used for the toxicity test.

### 2.2. Chemicals

The chemicals used in this toxicity study were 2-chloro-5-pyridinecarbaldehyde (CPC, C_6_H_4_ClNO, MW: 141.56, CAS No. 23100-12-1, Sigma-Aldrich, St. Louis, MO, USA) and 6-chloronicotinic acid (6-CNA, C_6_H_4_ClNO_2_, MW: 157.55, CAS No. 5326-23-8, Sigma-Aldrich).

### 2.3. Exposure Test

For the exposure test, the embryos were cultured in an embryo culture solution (10 mg/mL NaCl, 0.03 mg/mL KCl, 0.053 mg/mL CaCl_2_, and 0.034 mg/mL MgSO_4_, pH 7.3). The PTPWs were dissolved in the embryo culture solution at appropriate concentrations. The concentrations of the CPC were 5, 10, 15, and 20 mg/L and those of 6-CNA were 80 and 160 mg/L. In the CPC exposure experiment, embryos within 6 h of fertilization (day 0 embryos) were used. In the 6-CNA exposure experiment, in addition to day 0 embryos, embryos at 144 h post fertilization (day 7 embryos) were used to evaluate the effect of the length of the exposure on development and hatching. The reason for deciding to use 144 h post fertilization embryos as exposure targets was to evaluate the effects of exposed substances when the egg membrane softens prior to hatching. Medaka embryos were exposed to the respective chemicals of PTPWs at 25 °C, in a light/dark cycle: 16 h–8 h, with solution changes every 24 h. Table 1 shows the experimental conditions. Exposure concentrations were set based on the results of CPC swimming inhibition tests (acute toxicity value of 48 h EC50) for *Chironomus yoshimatsui* and *Daphnia magna* (*Chironomus yoshimatsui*: >5 mg/L, *Daphnia magna*: >34 mg/L) [26].

### 2.4. Observation

During the exposure test, the Medaka embryos were observed using a stereomicroscope (SMZ 800N, Nikon, Tokyo, Japan). Images of medaka embryos were obtained using an image acquisition application (Anyty Microscope, Three R Solution, Fukuoka, Japan). The endpoints of embryo observation were set as failure to develop, thrombus formation, malformations, and hatching.

## 3. Results

### 3.1. Effects of CPC on Embryonic Development

**Figure 1:**
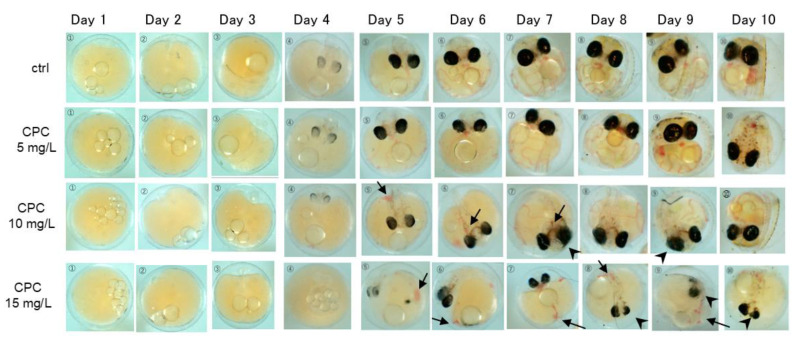
Developmental process of embryos exposed to 5, 10, and 15 mg/L CPC. No abnormalities were observed in the controls. Embryos exposed to 5 mg/L CPC developed normally as the controls. Embryos exposed to 10 mg/L CPC showed delayed development compared to the controls. Blood clots (arrows) and disorganized eyeballs (arrowheads) appeared in some embryos. Embryos had less blood flow compared to controls after day 6. In the 15 mg/L CPC group, development was severely delayed compared to controls. Thrombus formation was also observed more frequently than at 10 mg/L. On day 4, several embryos ceased to develop.

Figure 1 shows the developmental process of the embryos from day 1 to day 10 of exposure. In the controls, the formation of the spinal cord was observed on day 3, and the eyeballs and heart on day 4. On day 5, the eyeballs were clearly blackened and blood flow was observed. On day 6, a powerful heartbeat was observed, accompanied by an increase in the volume of blood flow and its momentum. From day 7 onward, body tissues were fully formed. On day 10, the embryos started hatching. In the CPC 5 mg/L exposure group, compared to the control embryos, there was no difference in the speed of development and no abnormalities in the appearance of the embryos. By day 10, 4 of the 15 embryos had hatched; by day 12, 11 embryos had hatched. On the other hand, in the embryos exposed to 10 mg/L CPC, one embryo exposed to 10 mg/L CPC stopped developing and died on day 4. On day 6 of exposure, developmental delays and thrombus formation were observed in one embryo. On day 7, one embryo had cardiac arrest and died. Thrombi were formed in embryos at day 5, 6, and 7 (Figure 1, arrows), and eyeball development was inhibited at days 7 and 9 (Figure 1, arrowheads). Finally, six embryos hatched by day 11. In the embryos exposed to 15 mg/L CPC, development was severely delayed compared to the controls, and thrombus formation was observed in more embryos than at 10 mg/L. On day 4, the development of several embryos had stopped. On day 7, there were six embryos that had formed thrombi.

**Figure 2:**
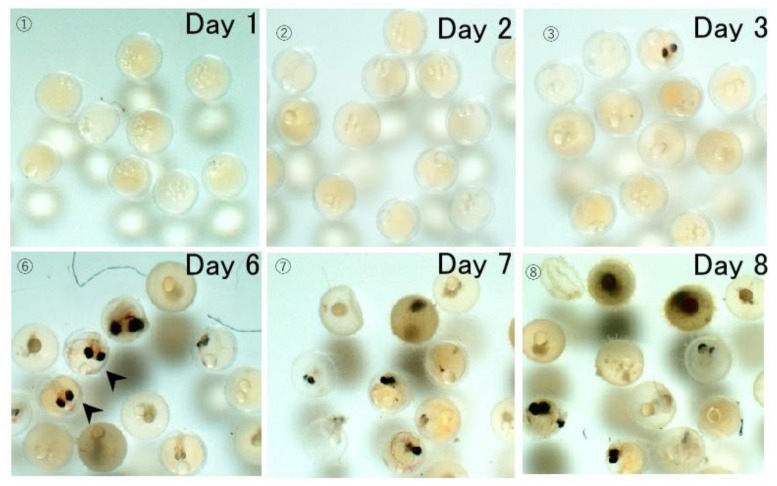
Developmental process of embryos exposed to 20 mg/L CPC. Compared to control embryos, the development was severely arrested. Retinal pigment epithelium was normally developed only in a few embryos by day 6 (arrowheads).

Figure 2 shows the developmental process of embryos exposed to 20 mg/L CPC. As mentioned above, the spinal cord and retinal pigment epithelium was formed in control embryos by days 3 and 5, respectively. However, the development of some embryos was completely arrested following treatment with 20 mg/L CPC. By day 6, 9 of the 15 embryos had stopped growing and died before their bodies were formed. By day 8, only one embryo was alive. As shown in Figure 2, pigment epithelium developed only in a few embryos (arrowheads). On day 8, thrombus formation was confirmed in most of the embryos in which blood vessels were formed. On day 7, the heart rate per 20 s was measured in one embryo in which a clot had formed. The value was 32.0 (σ = ±0.00), which was lower than 38.7 (σ = ±0.47) recorded in the control group.

### 3.2. Effects of 6-CNA on Embryonic Development

**Figure 3:**
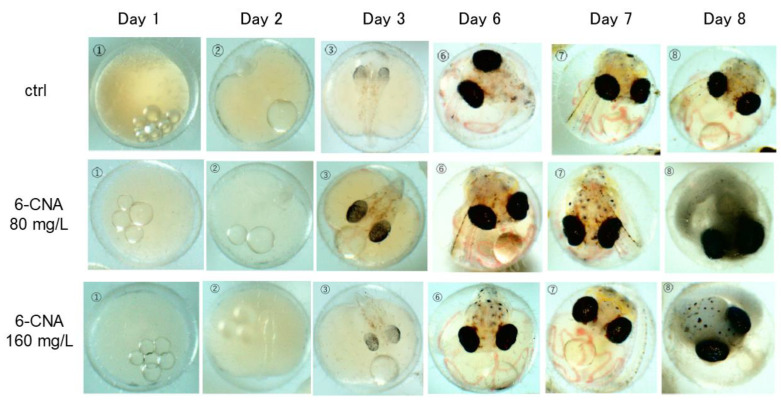
Developmental process of embryos exposed to 80 and 160 mg/L 6-CNA. In both groups, development proceeded normally until day 7, as retinal pigment epithelium and blood vessels were formed by day 3 and day 6, respectively.

Figure 3 shows the developmental process of embryos exposed to 80 and 160 mg/L 6-CNA. In contrast to CPC exposure, 6-CNA exposure did not cause developmental delay and abnormalities. The eyeballs were developed as in the controls and thrombus formation was not detected. However, at 80 mg/L 6-CNA, the embryos started to die at day 8, and 11 of 15 embryos died by day 9. At 160 mg/L 6-CNA, all 15 embryos died by day 7.

**Figure 4:**
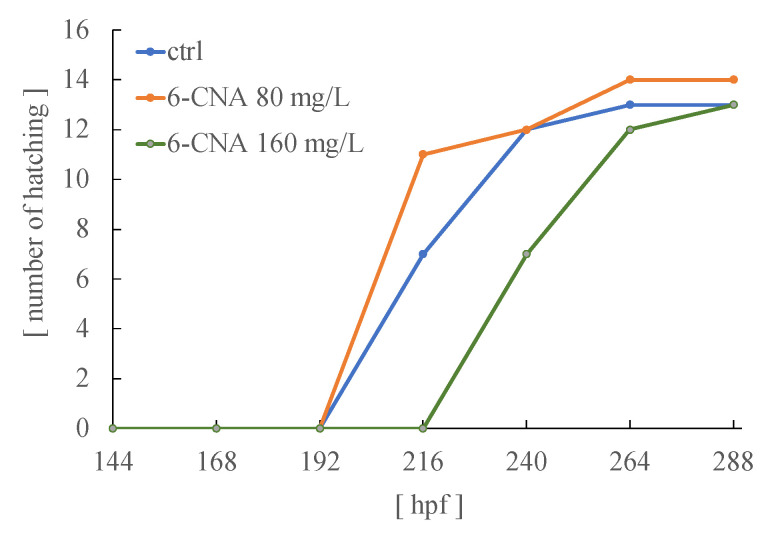
Number of hatched embryos exposed to 80 or 160 mg/L 6-CNA from day 7. When the embryos were exposed to 160 mg/L, but not 80 mg/L 6-CNA, hatching was delayed compared to the controls. Fifteen embryos were used in the experiment; however, two, one, and two embryos in the control, 80 mg/L, and 160 mg/L groups, respectively, did not hatch due to fungus on the eggs.

Figure 4 shows the number of hatched medaka embryos exposed to 80 and 160 mg/L 6-CNA. In this experiment, exposure was initiated on day 7. Medaka embryos in the control group started to hatch on day 9 (i.e., day 5 of exposure) and all 13 embryos had hatched by day 11. When the embryos were exposed to 80 mg/L 6-CNA, there were no abnormalities in the developmental process or hatching compared with the control. Hatching began on day 9 of exposure (5 days of exposure) and all embryos had hatched by day 11. On the other hand, embryos exposed to 160 mg/L showed delays in hatching. None of the embryos had hatched by day 9, and approximately 50% of embryos had hatched at day 10. However, no abnormalities of development were detected in these embryos.

## 4. Discussion

In the present study, thrombus formation, delayed development, and defective eyeball formation were observed in embryos exposed to CPC from day 0. These abnormalities were observed from day 5 in embryos exposed to 10 mg/L, from day 4 in those exposed to 15 mg/L, and from day 3 in those exposed to 20 mg/L, indicating that higher concentrations of CPC affected embryos from an earlier stage of development. Thrombus formation may be related to the effects of CPC on nicotinic acetylcholine receptors. The actual ligands for the acetylcholine receptors is acetylcholine. Exogenous nicotine will act as an agonist and may cause vasoconstriction and elevated blood pressure. Neonicotinoids such as imidacloprid act on nicotinic acetylcholine receptors in the same manner as nicotine [27]. Therefore, it is possible that CPC, a PTPWs of imidacloprid, acts on acetylcholine receptors in the same manner as imidacloprid, causing vasoconstriction and blood pressure elevation, which in turn causes thrombus formation. Furthermore, thrombus formation may prevent the flow of oxygenated blood to cells and tissues, resulting in cell necrosis and defective development of the eyes and other organs. Owing to CPC exposure, embryos with thrombus formation or eye abnormalities showed weaker heart beating than the controls. Nutrients from egg yolk are supplied to various body parts through the arterial blood to grow medaka embryos. It is likely that the weakening of the beat may have decreased blood circulation, thus reducing the amount of nutrients carried around the body and delaying embryo development.

When 6-CNA was administered to day 0 embryos, they died before hatching. Furthermore, when 160 mg/L 6-CNA was administered to day 7 embryos, the onset of hatching was delayed. During hatching, the inner layer of the chorion is chemically dissolved by hatching enzymes, and the embryo physically breaks weakened chorion. Hatching enzymes are synthesized by hatching glands. The synthesized hatching enzymes are stored in the cells of the glands and secreted during hatching. The development of hatching glands occurs in the early phase of development. In medaka, hatching glands begin to form 2–3 days after fertilization, and they are seen as a mass of large cells (<14 µm in diameter) by days 3–4. Enzymatic activity is observed by day 6 [28]. In this study, day 0 embryos exposed to 6-CNA did not hatch and died. When day 7 embryos were exposed to 6-CNA, they hatched adequately, although the onset of hatching was delayed. This suggests that 6-CNA does not affect the development of most of the organs but affects that of the hatching gland and/or synthesis or secretion of hatching enzymes. It is possible that in embryos exposed to 6-CNA from day 0, hatching glands were not normally formed or hatching enzymes could not be synthesized, leading to hatching defects. On the other hand, when the day 7 embryos were exposed to 6-CNA, hatching glands were already developed, and hatching enzymes should have started to be secreted by day 7; thus, the effect on hatching was considered to be minimal. However, even in this case, hatching was delayed, probably because synthesis of hatching enzymes was inhibited to some extent.

Herein, we revealed the lethal effects of CPC and 6-CNA, both of which are PTPWs of imidacloprid. Importantly, the effects of these compounds differed; CPC affected the development of most of the organs, but 6-CNA affected hatching gland development and/or hatching enzyme synthesis. In future, it is necessary to evaluate the actual binding of CPC and 6-CNA to acetylcholine receptors. In addition, the molecular mechanisms by which CPC and 6-CNA affect medaka development should also be investigated. This research will provide much insight and information regarding the effects of chemicals commonly used in our surroundings, such as pesticides, on non-target organisms. This is an important factor for chemical risk management. Moreover, the findings afford a convenient and comfortable life from the safe and secure use of chemicals, as well as the development of chemicals which are less likely to affect non-target organisms.

## 5. Conclusions

In this study, we revealed that CPC and 6-CNA, the PTPWs of imidacloprid, affect development of medaka embryos. Importantly, we showed that the mechanisms by which the development is damaged are different between CPC and 6-CNA: CPC causes developmental delay and malformation including thrombus formation and retinal pigment epithelium dysplasia. On the other hand, 6-CNA does not cause developmental delay and malformation, but inhibits hatching. In future, the molecular mechanisms by which CPC and 6-CNA affect medaka development should be addressed.

## Figures and Tables

**Table 1:** Exposure test conditions.

	Timing of Exposure	Sample Size
ctrl	<6 hfp	15
CPC 5 mg/L		
CPC 10 mg/L		
CPC 15 mg/L		
CPC 20 mg/L		
ctrl	<6 hfp	15
6-CNA 80 mg/L		
6-CNA 160 mg/L		
ctrl	144 hfp	15
6-CNA 80 mg/L		
6-CNA 160 mg/L		

hpf: hours post fertilization.

## Data Availability

Data are available upon reasonable request to the authors.
